# Biological Evaluation, Molecular Docking, and SAR Studies of Novel 2-(2,4-Dihydroxyphenyl)-1*H*- Benzimidazole Analogues

**DOI:** 10.3390/biom9120870

**Published:** 2019-12-12

**Authors:** Joanna Matysiak, Alicja Skrzypek, Monika Karpińska, Kamila Czarnecka, Paweł Szymański, Marek Bajda, Andrzej Niewiadomy

**Affiliations:** 1 Department of Chemistry, University of Life Sciences in Lublin, Akademicka 15, 20-950 Lublin, Poland; alicja.skrzypek@up.lublin.pl (A.S.); andrzej.niewiadomy@up.lublin.pl (A.N.); 2 Łukasiewicz Research Network – Institute of Industrial Organic Chemistry, Annopol 6, 03-236 Warsaw, Poland; karpinska@ipo.waw.pl; 3 Department of Pharmaceutical Chemistry, Drug Analyses and Radiopharmacy, Faculty of Pharmacy, Medical University of Lodz, Muszynskiego 1, 90-151 Lodz, Poland; kamila.czarnecka@umed.lodz.pl (K.C.); pawel.szymanski@umed.lodz.pl (P.S.); 4 Department of Physicochemical Drug Analysis, Faculty of Pharmacy, Jagiellonian University Medical College, Medyczna 9, 30-688 Cracow, Poland; marek.bajda@uj.edu.pl

**Keywords:** benzimidazole, acetylcholinesterase, butyrylcholinesterase, inhibitor, beta amyloid, molecular docking, lipophilicity, SAR

## Abstract

In the present study, new 4-(1*H*-benzimidazol-2-yl)-benzene-1,3-diols, modified in both rings, have been synthesized and their efficacies as acetylcholinesterase (AChE) and butyrylcholinesterase (BuChE) inhibitors have been determined. The modified Ellman’s spectrophotometric method was applied for the biological evaluation. The compounds showed strong (IC_50_ 80–90 nM) AChE and moderate (IC_50_ 5–0.2 µM) BuChE inhibition in vitro. Some compounds were effective toward AChE/BuChE, exhibiting high selectivity ratios versus BuChE, while the other compounds were active against both enzymes. The structure–activity relationships were discussed. The compounds inhibited also in vitro self-induced Aβ(1–42) aggregation and exhibited antioxidant properties. The docking simulations showed that the benzimidazoles under consideration interact mainly with the catalytic site of AChE and mimic the binding mode of tacrine.

## 1. Introduction

The therapy of neurodegenerative diseases, including Alzheimer’s disease (AD), is a great challenge in the modern world [1]. Both acetylcholinesterase (AChE) and butyrylcholinesterase (BuChE) enzymes are promising molecular targets for AD drug design [2]. Many different heterocyclic systems are explored in this field including the benzimidazole skeleton [3,4,5,6]. The amine group is an integral part of the structure of numerous designed AChE inhibitors [4]. 

**Figure 1:**
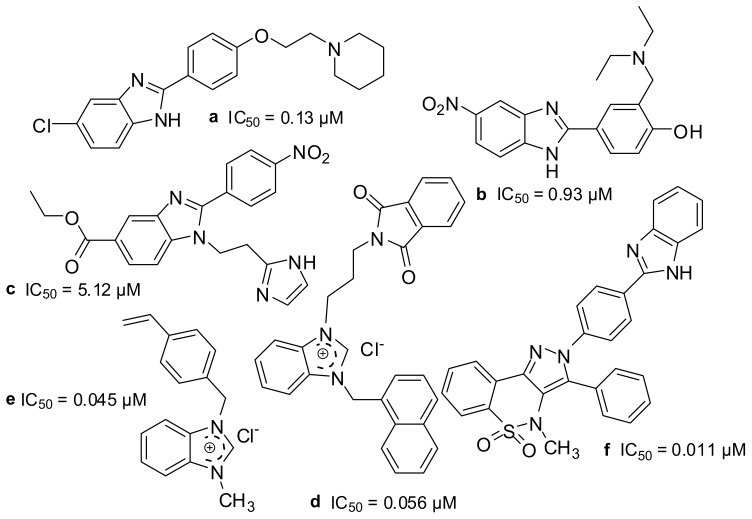
Structures of benzimidazole analogs as AChE inhibitors.

*N*-substituted 2-aminobenzimidazoles were described as good inhibitors of AChE and BuChE. The molecular modeling studies revealed that the heterocyclic ring of the active molecule creates face-to-face π–π stacking interactions in the form of a “sandwich” with the indole moiety of the appropriate amino acids [5]. N-differently substituted amine derivatives of 2-arylbenzimidazoles (compound **a**, Figure 1) were presented by Alpan et al. [6]. It was found that the compounds exhibited significant AChE and/or BuChE inhibitory activities (IC_50_ = 0.6–0.1 µM) and some of them were selective against AChE/BuChE. The Mannich bases of 2-(4-hydroxyphenyl)benzimidazole derivatives (e.g., compound **b**, Figure 1) display moderate to good AChE inhibitory potency with IC_50_ ranging from 0.93 to 10 µM as well as moderate BuChE inhibitory activity. The molecular modeling studies showed that, among others, the 4-hydroxyl group participated in the crucial interaction with AChE [7]. Moderate BuChE inhibitors were found in a group of 2-[4-(4-substitutedpiperazin-1-yl)phenyl]benzimidazoles. The most active compounds showed an inhibitory effect on the self-mediated amyloid β-peptide(1–40) (Aβ(1–40)) aggregation [8]. Free of amine group benzimidazole derivatives were also presented as AChE/BuChE inhibitors. They include 2-(chloro/nitro)arylbenzimidazoles [9] and *N*-substituted-2-aryl-1*H*-benzimidazole-5-carboxylates [10]. The highest activity against AChE was found for 1-(3-(1*H*-imidazol-1-yl)propyl)- 2-(4-nitrophenyl)-1*H*-benzimidazole-5-carboxylate (compound **c**, Figure 1) [10]. The other researchers studied benzimidazole salts. A nanomolar activity was found for *N*-propylphthalimide- and 4-vinylbenzyl-substituted benzimidazole salts (compounds **d** and **e**, Figure 1) [11]. A large group of imidazolium and benzimidazolium oximes was designed for reactivation of phosphylated human BuChE [12]. A prominent (nanomolar) activity against AChE/BuChE was found for the pyrazolobenzothiazine-benzimidazole hybrids (compound **f**, Figure 1) [13] and the benzimidazole-based organophosphorus compounds [14]. Most of the mentioned compounds are the multitargeted agents: carbonic anhydrase inhibitors [11], antioxidants [7], interrupters of cell cycle [9], Fe^2+^ ion chelators [11]. Some of them show anticancer potency [9,15] or neuroprotective properties [8]. Benzimidazole derivatives are also known as β-glucuronidase [16,17], α-glucosidase [18,19], urease [20], and α-amylase enzymes inhibitors [21]. 

Molecular modeling studies for 5-aryl-4-(1,3,4-thiadiazol-2-yl)benzene-1,3-diols obtained by us indicated a network of essential bonds between the -OH groups of the benzenediol ring and the residues of amino acid AChE in the active site which results in a significant inhibitory effect against AChE [22,23]. Some of them also displayed neuroprotective properties [24]. Therefore, a decision was made to obtain new benzimidazoles with 2,4-dihydroxyphenyl moiety as potential AChE/BuChE inhibitors. Searching for the optimal substitution of the benzenediol ring, the third group -OH or the hydrophobic substituent (-Cl, -Me, -Et) was introduced. In the heterocyclic ring, there were taken into consideration, among others, the substituents -OMe and -CF_3_ which intensified the inhibition activity of 1,3,4-thiadiazole derivatives.

Previously studied 4-(1*H*-benzimidazol-2-yl)benzene-1,3-diols and their metal complexes showed significant anticancer and antimicrobial potencies [25,26,27,28]. Polyphenol derivatives are commonly known as antioxidants. Therefore, this is justified to create benzimidazole–resorcinol conjugates with the additional potential antioxidant properties. It is also known that oxidative stress has a significant impact on the pathogenesis of neurodegenerative diseases [2,29,30].

The paper presents the synthesis of new 1*H*-benzimidazoles functionalized by the resorcinol moiety and evaluation of their inhibitory activities against cholinesterases, as well as inhibition mechanism studies, molecular docking, chromatography lipophilicity estimation, and structure–activity elucidation. The evaluation of in vitro self-induced Aβ(1–42) aggregation inhibition and antioxidant properties were also performed. 

## 2. Materials and Methods 

### 2.1. Analytical Studies

Melting points (m.p.) were determined using a BÜCHI B-540 (Flawil, Switzerland) melting point apparatus and they were uncorrected. The elemental analysis (C, H, N) was performed on Perkin-Elmer 2400. The elemental analysis was within 0.5% of the theoretical value. The purity of compounds was monitored by reverse-phase high-performance liquid chromatography (RP-HPLC), RP-18/MeOH/H_2_O chromatography. The MS spectra (EI, 70 eV) were recorded using the apparatus AMD-604. The IR spectra were registered on the Perkin-Elmer FT-IR 1725X spectrophotometer (in KBr) in the range of 600–4000 cm^−1^. The NMR spectra (1D, ^1^H NMR, and ^13^C NMR) were recorded in DMSO-*d*_6_ using a Bruker DRX 500 (Bruker Daltonics, Inc. Billerica, MA, USA). Chemical shifts (δ, ppm) were described in relation to tetramethylsilane (TMS) and the coupling constants (J) were expressed in Hz. 

### 2.2. Synthesis of the Compounds

The general procedure for the synthesis of the compounds is as follows. A mixture of the corresponding benzene-1,2-diamine (3 mmol) and the corresponding sulfinylbis[(2,4-dihydroxyphenyl)- methanethione] (**STB**) (3 mmol) in MeOH (20 mL) was heated to reflux for 3–4 h. The hot mixture was filtered. Following, the filtrate was concentrated or left at room temperature. Finally, the product was crystalized from the MeOH or MeOH/H_2_O solution.

*4-(1H-benzimidazol-2-yl)-6-chlorobenzene-1,3-diol* (**1**). Yield: 84%; m.p.: 340–341 °C; EI MS (*m*/*z*, %): 260 (M^+^, 100), 231 (6), 197 (17), 169 (25), 143 (5), 116 (6), 92 (5), 69 (4), 65 (7), 63 (5), 52 (3), 36 (17); ^1^H NMR (500 MHz, DMSO-*d_6_*) δ: 14.12 (s, 1H, NH), 11.46 (s, 1H, C(3)–OH), 10.44 (s, 1H, C(1)–OH), 8.27 (s, 1H, C(5)–H), 7.80 (m, 2H, C(4′,7′)–H), 7.49 (m, 2H, C(5′,6′)–H), 7.03 (s, 1H, C(2)–H) ppm; ^13^C NMR (125 MHz, DMSO-*d_6_*) δ: 161.1, 157.3, 152.3, 140.3 (2C), 129.3, 123.2 (2C), 114.8 (2C), 112.6, 111.9, 103.1 ppm; IR (KBr, cm^−1^): 3281 (OH), 3051 (C–H), 1608 (C=N), 1572 (C=N), 1518 (C=C), 1503 (C=C), 1460, 1403, 1390, 1344, 1308, 1285, 1243, 1229 (C–OH), 1215, 1153, 1098, 1041, 1003, 951, 895, 849, 816, 779, 757, 737, 704. Anal. Calc. for C_13_H_9_ClN_2_O_2_ (260.68): C, 59.90; H, 3.48; N, 10.75. Found: C, 59.94; H, 3.51; N, 10.68%.

*4-(5,6-Dichloro-1H-benzimidazol-2-yl)-6-chlorobenzene-1,3-diol* (**2**). Yield: 88%; m.p.: 354–355 °C; EI MS (*m*/*z*, %): 329 (M^+^, 100), 296 (18), 256 (14), 201 (50), 187 (21), 169 (14), 146 (6), 141 (8), 114 (7), 95 (2), 86 (3), 73 (9), 69 (8), 63 (15), 51 (5), 45 (3), 40 (9); ^1^H NMR (500 MHz, DMSO-*d_6_*) δ: 11.20 (s, 1H, C(3)–OH), 10.78 (s, 1H, C(1)–OH), 8.11 (s, 1H, C(5)–H), 7.52 (s, 2H, C(4′,7′)–H), 6.72 (s, 1H, C(2)–H) ppm; IR (KBr, cm^−1^): 3496 (OH), 3354 (NH), 3071 (OH), 1640 (C=N), 1594 (C=N), 1512 (C=C), 1471, 1422, 1399, 1299, 1271, 1211 (C–OH), 1197, 1161, 1089, 1062, 967, 875, 846, 832, 803, 774, 748, 731. Anal. Calc. for C_13_H_7_Cl_3_N_2_O_2_ (329.57): C, 47.38; H, 1.14; N, 8.50. Found: C, 47.29; H, 2.17; N, 8.57%.

*4-[6-(Trifluoromethyl)-1H-benzimidazol-2-yl]-2-methylbenzene-1,3-diol* (**3**). Yield: 72%; m.p.: 263–264 °C; EI MS (*m*/*z*, %): 308 (M^+^, 100), 289 (7), 279 (37), 263 (12), 251 (9), 237 (7), 211 (4), 167 (61), 154 (7), 140 (5), 65 (5), 39 (4); ^1^H NMR (500 MHz, DMSO-*d_6_*) δ: 13.07 (s, 1H, C(3)–OH), 10.54 (s, 1H, C(1)–OH), 8.17 (d, *J* = 8.9 Hz, 1H, C(5)–H), 7.85 (s, 1H, C(7′)–H), 7.71 (dd, *J* = 8.6 and 1.4 Hz, 1H, C(5′)–H), 6.65 (d, *J* = 8.5 Hz, 1H, C(4′)–H), 6.59 (d, *J* = 8.9 Hz, 1H, C(6)–H), 2.00 (s, 3H, Me) ppm; IR (KBr, cm-1): 3378 (OH, NH), 2924 (CH), 1611 (C=N), 1573 (C=C), 1504 (C=C), 1455, 1377, 1336, 1305, 1267 (C–OH); 1194, 1131, 1111, 1077, 1037, 930, 856, 832, 822, 793, 756, 705. Anal. Calc. for C_15_H_11_F_3_N_2_O_2_ (308.26): C, 58.45; H, 3.60; N, 9.09. Found: C, 58.52; H, 3.58; N, 9.16%.

*4-(7-Chloro-5-(trifluoromethyl)-1H-benzimidazol-2-yl)-6-ethylbenzene-1,3-diol* (**4**). Yield: 81%; m.p.: 284–286 °C; EI MS (*m*/*z*, %): 357 (M^+^ + 1, 100), 356 (M^+^, 28), 341 (M^+^ – Me, 70), 313 (4), 287 (9), 271 (8), 212 (5), 181 (46), 164 (16), 149 (3), 77 (5), 69 (11), 45 (2), 39 (3); ^1^H NMR (500 MHz, DMSO-*d_6_*) δ: 11.30 (s, 1H, NH), 10.75 (s, 1H, C(3)–OH), 10.04 (s, 1H, C(1)–OH), 7.75 (s, 1H, C(4′)–H), 7.58 (s, 1H, C(5)–H), 7.43 (s, 1H, C(6′)–H), 6.44 (s, 1H, C(2)–H)), 2.46 (q, *J* = 7.4 Hz, 2H, CH_2_Me), 1.13 (t, *J* = 7.4 Hz, 3H, CH_2_Me) ppm; IR (KBr, cm^−1^): 3456 (OH), 3402 (OH), 3356 (NH), 2971 (CH), 2939 (CH), 2874 (CH), 1624 (C=N), 1594 (C=C), 1508 (C=C), 1488, 1467, 1346, 1291, 1248 (C–OH), 1146, 1094, 1071, 987, 916, 887, 844, 768, 748. Anal. Calc. for C_16_H_12_ClF_3_N_2_O_2_ (356.73): C, 53.87; H, 3.39; N, 7.58. Found: C, 53.94; H, 3.37; N, 7.62%.

*4-(6-Methoxy-1H-benzimidazol-2-yl)-2-methylbenzene-1,3-diol* (**5**). Yield: 76%; m.p.: 228–229 °C; EI MS (*m*/*z*, %): 270 (M^+^, 100), 255 (57), 241 (11), 227 (13), 198 (3), 167 (6), 151 (4), 135 (8), 79 (3), 77 (4), 65 (2), 44 (7), 39 (3), 33 (5); ^1^H NMR (500 MHz, DMSO-*d_6_*) δ: 13.37 (s, 1H, C(3)–OH), 10.42 (s, 1H, C(1)–OH), 8.15 (d, *J* = 8.9 Hz, 1H, C(5)–H), 7.38 (d, *J* = 8.9 Hz, 1H, C(4’)–H), 7.03 (dd, *J* = 8.9 Hz and 2.8 Hz, 1H, C(5′)–H), 6.98 (d, *J* = 2.74 Hz, 1H, C(7′)–H), 6.57 (d, *J* = 7.9 Hz, 1H, C(6)–H), 3.30 (s, 3H, OMe), 2.00 (s, 3H, Me) ppm; ^13^C NMR (125 MHz, DMSO-*d_6_*) δ: 161.1, 159.2, 156.9, 156.0, 154.6, 139.5, 138.1, 126.2, 114.8, 111.2, 108.3, 108.8, 102.9, 59.8, 8.2 ppm; IR (KBr, cm^−1^): 3382 (OH), 2937 (CH), 1611 (C=N), 1571 (C=C),1502 (C=C), 1457, 1335, 1319, 1252, 1236 (C–OH), 1183, 1159, 1108, 1091, 1036, 955, 916, 857, 815, 777, 758, 738, 700. Anal. Calc. for C_15_H_14_N_2_O_3_ (270.16): C, 66.66; H, 5.22; N, 10.36. Found: C, 66.74; H, 5.24; N, 10.29%.

*4-(6-Methoxy-1H-benzimidazol-2-yl)-6-chlorobenzene-1,3-diol* (**6**). Yield: 73%; m.p.: 219–222 °C; EI MS (*m*/*z*, %): 290 (M^+^, 100), 275 (82), 261 (5), 247 (20), 241 (4), 187 (6), 145 (6), 80 (4), 78 (3), 69 (3), 51 (3), 33 (6); ^1^H NMR (500 MHz, DMSO-*d_6_*) δ: 12.77 (s, 1H, C(3)–OH), 11.40 (s, 1H, C(1)–OH), 8.19 (s, 1H, C(5)–H), 7.41 (d, *J* = 8.8 Hz, 1H, C(4′)–H), 7.06 (dd, *J* = 8.9 and 2.8 Hz, 1H, C(5′)–H), 7.02 (d, *J* = 2.8 Hz, 1H, C(7′)–H), 6.56 (s, 1H, C(2)–H), 3.27 (s, 3H, CH_3_) ppm; ^13^C NMR (125 MHz, DMSO-*d_6_*) δ: 161.3, 157.1, 156.2, 154.9, 139.1, 134.3, 116.2, 114.7, 111.9, 111.5, 108.2, 104.2, 102.9, 59.8 ppm; IR (KBr, cm^−1^): 3403 (NH), 3198 (OH), 2951 (CH), 1633 (C=N), 1512 (C=C), 1476, 1328, 1228 (C–OH), 1187, 1137, 1109, 1090, 974, 883, 784, 756, 727. Anal. Calc. for C_14_H_11_ClN_2_O_3_ (290.70): C, 57.84; H, 3.81; N, 9.64. Found: C, 57.92; H, 3.78; N, 9.70%.

*4-(6-Methoxy-1H-benzimidazol-2-yl)benzene-1,2,3-triol* (**7**) [31].

*Ethyl 2-(2,4-dihydroxy-3-methylphenyl)-1H-benzimidazole-5-carboxylate* (**8**). Yield: 73%; m.p.: 213–214 °C; EI MS (*m*/*z*, %): 312 (M^+^, 100), 284 (31), 267 (15), 255 (13), 239 (11), 167 (5), 119 (5), 90 (3), 63 (3); ^1^H NMR (500 MHz, DMSO-*d_6_*) δ: 12.21 (s, 1H, NH), 10.91 (s, 1H, C(2)–OH), 10.06 (s, 1H, C(4)–OH), 7.66 (dd, *J* = 8.6 and 1.9 Hz, 1H, C(6′)–H), 7.62 (d, *J* = 8.9 Hz, 1H, C(5)–H), 7.56 (s, 1H, C(4′)–H), 6.79 (d, *J* = 8.57 Hz, 1H, C(7′)–H), 6.42 (d, *J* = 8.84 Hz, 1H, C(6)–H), 3.75 (q, *J* = 7.01 Hz, 2H, CH_2_Me), 1.82 (s, 3H, Me), 1.38 (t, *J* = 7.0 Hz, 3H, CH_2_Me) ppm; IR (KBr, cm^−1^): 3419 (OH), 3328 (NH), 3204 (OH), 2920 (CH), 1673 (C=O), 1627 (C=N), 1602 (C=C), 1493, 1459, 1394, 1369, 1294, 1263, 1242 (C–OH), 1202, 1155, 1088, 1015, 966, 857, 821, 796, 765, 726. Anal. Calc. for C_17_H_16_N_2_O_4_ (312.32): C, 65.38; H, 5.16; N, 8.97. Found: C, 65.31; H, 5.19; N, 8.90%.

*Ethyl 2-(5-ethyl-2,4-dihydroxyphenyl)-1H-benzimidazole-5-carboxylate* (**9**). Yield: 74%; m.p.: 316–317 °C; EI MS (*m*/*z*, %): 326 (M^+^, 70), 311 (M^+^ − Me, 100), 297 (10), 283 (48), 238 (10), 169 (3), 133 (12), 123 (9), 91 (4), 44 (4), 36 (5); ^1^H NMR (500 MHz, DMSO-*d_6_*) δ: 10.71 (s, 1H, C(2)–OH), 8.33 (s, 1H, C(6)–H), 8.03 (d, *J* = 8.5 Hz, 1H, C(6′)–H), 7.84 (s, 1H, C(4′)–H), 7.45 (d, *J* = 8.5 Hz, 1H, C(7′)–H), 6.74 (s, 1H, C(3)–H), 4.38 (q, *J* = 7.5 Hz, 2H, OCH_2_Me), 2.50 (q, *J* = 7.1 Hz, 2H, CH_2_Me), 1.37 (t, *J* = 7.1 Hz, 3H, CH_2_Me), 1.21 (t, *J* = 7.5 Hz, 3H, OCH_2_Me) ppm; IR (KBr, cm^−1^): 3425 (NH), 3163 (OH), 2968 (CH),1677 (C=O), 1618 (C=N), 1569 (C=C), 1511 (C=C), 1466, 1408, 1373, 1296, 1245 (C–OH), 1149, 1094, 1014, 951, 837, 765, 742. Anal. Calc. for C_18_H_18_N_2_O_4_ (326.35): C, 66.25; H, 5.56; N, 8.58. Found: C, 66.31; H, 5.55; N, 8.51%.

*Ethyl 2-(2,4,6-trihydroxyphenyl)-1H-benzimidazole-5-carboxylate* (**10**). Yield: 63%; m.p.: 300–301 °C; EI MS (*m*/*z*, %): 314 (M^+^, 36), 286 (9), 269 (9), 250 (10), 230 (21), 222 (22), 206 (6), 194 (13), 185 (6), 177 (18), 149 (8), 126 (100), 111 (7), 97 (8), 90 (5), 85 (22), 80 (13), 69 (23), 63 (10), 52 (12), 44 (27), 39 (10), 36 (7); ^1^H NMR (500 MHz, DMSO-*d_6_*) δ: heteroatoms protons are illegible; 8.02 (m, 2H, CH), 7.65 (m, 1H, CH), 5.91 (s, 1H, CH), 5.89 (s, 1H, CH), 4.35 (q, *J* = 7.0 Hz, 2H, CH_2_Me), 1.35 (t, *J* = 7.1 Hz, 3H, CH_2_Me) ppm; IR (KBr, cm^−1^): 3435 (OH, NH), 1667 (C=O), 1615 (C=N), 1517 (C=C), 1478, 1390, 1366, 1289, 1244 (C–OH), 1189, 1156, 1028, 934, 823, 766, 729. Anal. Calc. for C_16_H_14_N_2_O_5_ (314.29): C, 61.14; H, 4.49; N, 8.91. Found: C, 61.08; H, 4.52; N, 8.85%.

*2-(2,4-Dihydroxy-3-methylphenyl)-1H-benzimidazole-6-carbonitrile* (**11**). Yield: 76%; m.p.: 338–339 °C; EI MS (*m*/*z*, %): 265 (M^+^, 100), 236 (32), 220 (10), 208 (10), 194 (6), 181 (3), 168 (3), 117 (4), 90 (4), 36 (4); ^1^H NMR (500 MHz, DMSO-*d_6_*) δ: 10.07 (s, 1H, C(1)–OH), 8.11 (s, 1H, C(7′)–H), 7.75 (m, 2H, CH), 7.65 (dd, *J* = 8.3 and 1.3 Hz, 1H, C(5′)–H), 6.57 (d, *J* = 8.7 Hz, 1H, C(6)–H), 2.07 (s, 3H, Me) ppm; IR (KBr, cm^−1^): 3369 (NH), 3134 (OH), 2837 (CH), 1659 (C=N), 1605 (C=C), 1570 (C=C), 1474, 1413, 1369, 1317, 1273, 1235 (C–OH), 1213, 1087, 981, 874, 819, 756, 710. Anal. Calc. for C_15_H_11_N_3_O_2_ (265.27): C, 67.92; H, 4.18; N, 15.84. Found: C, 68.01; H, 4.20; N, 15.77%.

*2-(5-Ethyl-2,4-dihydroxyphenyl)-1H-benzimidazole-6-carbonitrile* (**12**). Yield: 84%; m.p.: 284–285 °C; EI MS (*m*/*z*, %): 279 (M^+^, 40), 264 (M^+^ − Me, 100); 236 (5), 207 (4), 194 (7), 181 (3), 117 (3), 90 (3), 40 (2), 36 (3); ^1^H NMR (500 MHz, DMSO-*d_6_*) δ: heteroatoms protons are illegible; 8.18 (s, 1H, C(7′)–H), 7.89 (s, 1H, C(6)–H), 7.84 (d, *J* = 8.3 Hz, 1H, C(4′)–H), 7.77 (m, 1H, C(5′)–H), 6.66 (s, 1H, C(3)–H), 2.45 (q, *J* = 7.5 Hz, 2H, CH_2_Me), 1.20 (t, *J* = 7.5 Hz, 3H, CH_2_Me) ppm. Anal. Calc. for C_16_H_13_N_3_O_2_ (279.29): C, 68.81; H, 4.69; N, 15.05. Found: C, 68.72; H, 4.71; N, 14.98%.

*4-(6-Nitro-1H-benzimidazol-2-yl)benzene-1,3-diol* (**13**) [31]. 

*2-Methyl-4-(6-nitro-1H-benzimidazol-2-yl)benzene-1,3-diol* (**14**). Yield: 74%; m.p.: 213–215 °C; EI MS (*m*/*z*, %): 285 (M^+^, 100), 256 (14), 239 (55), 210 (16), 167 (13), 151 (11), 119 (5), 90 (8), 77 (6), 63 (11), 52 (5), 39 (5), 33 (3); ^1^H NMR (500 MHz, DMSO-*d_6_*) δ: 12.15 (s, 1 H, NH), 10.99 (s, 1H, C(3)–OH), 10.15 (s, 1H, C(1)–OH), 7.98 (dd, *J* = 9.1 Hz and 2.6 Hz, 1H, C(5′)–H), 7.92 (d, *J* = 2.6 Hz, 1H, C(7′)–H), 7.65 (d, *J* = 8.6 Hz, 1H, C(5)–H), 6.82 (d, *J* = 9.1 Hz, 1H, C(4′)–H), 6.47 (d, *J* = 8.8 Hz, 1H, C(6)–H), 2.02 (s, 3H, Me) ppm; ^13^C NMR (125 MHz, DMSO-*d_6_*) δ: 195.1, 158.3, 151.3, 135.3, 133.2, 126.2, 124.8, 123.4, 122.6, 117.1, 111.3, 110.1, 107.4, 8.4 ppm; IR (KBr, cm^−1^): 3426 (NH), 3340 (OH), 3222 (OH), 2855 (CH), 1641 (C=N), 1604 (C=C), 1514 (C=C), 1499, 1462, 1428, 1382, 1382, 1307, 1262 (C–OH), 1203, 1153, 1080, 887. Anal. Calc. for C_14_H_11_N_3_O_4_ (285.25): C, 58.95; H, 3.89; N, 14.73. Found: C, 59.03; H, 3.91; N, 14.68%.

### 2.3. RP-18 HPLC Chromatography

The Eurosil Bioselect C18 (5 μm, 300 × 4.6 mm) column was used as the stationary phase. The mobile phase consisted of different volume mixtures of methanol and 20 mM acetate buffer as the aqueous phase to give pH 4.0. The organic modifier concentration ranged from 0.30 to 0.75, depending on the structure of the compound at 0.05 (0.1) intervals. The flow rate was 0.5 cm^3^·min^−1^ at room temperature. Measurements were made at 292 nm. The retention time of an unretained solute (t_0_) was determined by the injection of a small amount of acetone dissolved in water. All compounds showed the regular retention behavior in the function of methanol concentration (*v*/*v*). This is described by the Soczewiński–Wachtmeister dependence:

log k = log k_w_ + S (% organic modifier) (1)

in which log k_w_ is the retention factor of a solute with pure water as the mobile phase (intercept) and S is the regression curve (slope) [32,33]. The log k values were calculated as log k = log (t_R_ − t_0_)/t_0_, where t_R_ is the retention time of a solute and t_0_ is the retention time of an unretained solute. 

### 2.4. In Vitro AChE and BuChE Inhibition Assay

Acetylcholinesterase (AChE, E.C. 3.1.1.7, from human erythrocytes), butyrylcholinesterase (BuChE, E.C. 3.1.1.8, from equine serum), acetylthiocholine iodide (ATCh), butylthiocholine iodide (BTCh), 5,5′-dithiobis-(2-nitrobenzoic acid) (DTNB), donepezil, and neostigmine bromide were purchased from Sigma-Aldrich (Steinheim, Germany). The inhibitory activities against AChE and BuChE of the prepared compounds were performed by means of the method previously developed by Ellman et al. using neostigmine and donepezil as the reference compound [34]. The details were described previously [22].

### 2.5. Kinetic Characterization of AChE and BuChE Inhibition

The kinetic analysis was evaluated according to the Ellman’s method [34]. Plots of 1/velocity versus the reciprocal substrate concentration were constructed at different final concentrations of the ATC iodide in the range of 100–10 µM. The studies were carried out using the final concentrations of **1** inhibitor: (AChE: 0.25, 1.0 µM; BuChE: 0.25, 1.2 µM) and **5** (AChE: 0.1, 1.0 µM; BuChE: 0.25, 0.55 µM) and without it. The tested medium contained DTNB solution (0.4 mg/mL), AChE solution (2 U/mL) or BuChE solution (4 U/mL), solution of the examined compound, and different concentrations of the ATC iodide substrate solution. Absorbance was detected at 412 nm at room temperature after 7 min. The obtained data were calculated and presented on the Lineweaver–Burk plot. The determined the Michaelis-Menten constant (K_M_) and the maximum velocity of the enzyme (υ_max_) values were used to define the type of enzyme inhibition.

### 2.6. Determination of the Inhibitory Effect on the Self-Mediated Aβ(1–42) Aggregation

Amyloid β-peptide, (1–42) (Aβ42), was purchased from Aldrich. Aβ42 was diluted in the phosphate buffer (pH 8.0) to obtain a 125.8 µM solution. The compounds under study were first dissolved in a small amount of methanol, and then further diluted in the phosphate buffer to the final concentration of 5–160 μM. To study the Aβ42 aggregation inhibition, the fluorescence emission of thioflavin T (ThT) was followed with curcumin as the reference compound [35,36]. At first, the Aβ42 (10 μL) samples and the tested compounds (10 μL) were diluted with the phosphate buffer (60 μL) and then they were incubated for 24 h at 37 °C without stirring. As for the control, a sample of the peptide was incubated without the inhibitor. The tests were carried out in black, flat-bottom, 96-well plates. Then 20 µL of 2.5 µM ThT in 50 mM glycine–NaOH (pH 8.5) was added and incubated for 5 min. Fluorescence was monitored with the excitation at 446 nm and the emission at 490 nm. A time scan of fluorescence was performed and the intensity values reached the plateau (300 s). The percent inhibition was calculated from:

I% = 100 − (IF_I_/IF_0_ * 100) (2)

in which IF_I_ and IF_0_ correspond to the fluorescence intensities, in the presence and absence of the test compounds, respectively, minus the fluorescence intensities due to the blanks. The reported values were obtained as the mean ± SD of triplicate of three independent experiments. 

### 2.7. In Vitro Antioxidant Activity Assay

Antioxidant activity of each compound and standards (rutin) was assessed based on the radical scavenging effect of stable DPPH free radical [37]. An amount of 10 μL of each tested compound or standard series of different concentrations (from 0.0 to 100 µM) was added to 9 mL of methanol solution of 2,2-diphenyl-1-picrylhydrazyl (DPPH) (100 μM). After incubation at 37 °C for 30 min, decrease in the absorbance of each solution was measured at 520 nm. The absorbance of the blank sample containing the same amount of DMSO and DPPH solution was also prepared and measured. All experiments were conducted in triplicate. The scavenging potential was compared with a solvent control (0% radical scavenging) and the standard compound. Radical scavenging activity (%AA) was calculated by the following formula:

(%AA) = [(A_0_ − A_1_)/A_0_] × 100% (3)

in which (A_0_) is the absorbance of the blank sample and (A_1_) is the absorbance of the tested compound (t = 30 min). Concentration of each compound required for scavenging 50% of DPPH (IC_50_) was also determined [38].

### 2.8. Computational Methods

The partition coefficient (log P) values were predicted using Crippen′s fragmentation by the CS ChemProp module from ChemDraw Ultra 12 (CambridgeSoft, Cambridge, MA, USA) according to the fragmentation method introduced by Crippen [39]. The polar surface area (tPSA) was calculated by the atom-based method [40].

### 2.9. Molecular Modeling Studies

The spatial structures of compounds **1**, **5**, and **7** were created by Corina online (Molecular Networks) and later prepared by Sybyl 8.0 (Tripos). All atom and bond types were checked, necessary hydrogen atoms were added, and finally, the Gasteiger–Marsili charges were assigned. The pK_a_ values for the compounds and the ionization states corresponding to the physiological conditions (pH 7.4) were assessed by Marvin (ChemAxon). The compounds were docked to acetylcholinesterase from the 1ACJ crystal structure. Before docking with GoldSuite (CCDC), the enzyme structure was prepared. All histidine residues were protonated at Nε, the hydrogen atoms were added, and some water molecules (616, 634, 643) were retained. The binding site was defined as all amino acid residues within the radius of 12 Å from the reference compound, tacrine. A standard set of genetic algorithms was applied. The population size was equal to 100, and the number of operations 100,000. As a result, 10 conformations for each compound were obtained. The GoldScore function was used for the selection of the best poses. The results were visualized by PyMOL 0.99 (DeLano Scientific LLC, Palo Alto, CA, USA).

## 3. Results and Discussion

### 3.1. Chemistry

**Scheme 1:**
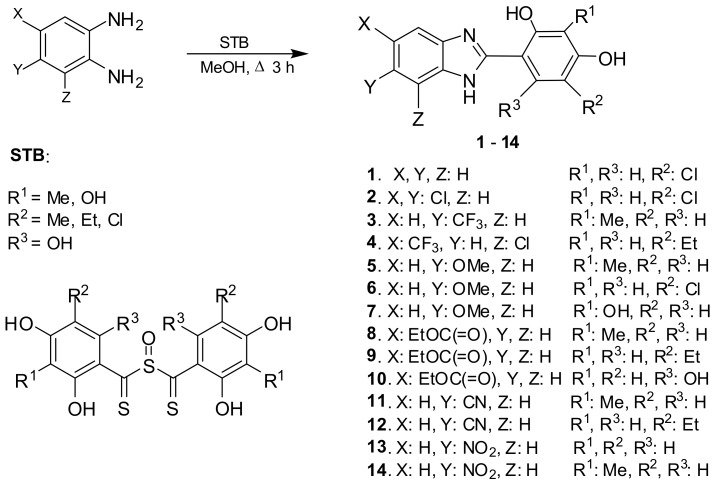
Synthesis of 2-(2,4-dihydroxyphenyl)-1*H*-benzimidazoles.

The target benzimidazole derivatives (Scheme 1) have been obtained in a one-step procedure using benzene-1,2-diamines with sulfinylbis[(2,4-dihydroxyphenyl)methanethione] (**STB**) or its analogs substituted by -Me, -Et, -Cl, or -OH on the resorcinol moiety in the reaction. These compounds are the agents cyclizing and introducing resorcinol (or modified one) into final products. They were obtained according to the previously described way [41]. The synthesis procedure was conducted for 2–4 h and the compounds were obtained in a good yield (60–87%). The product purity was evaluated by RP-HPLC (C-18, methanol–water mobile phase). The synthetic scheme and structures of benzimidazoles are presented in Scheme 1. 

The structures of the compounds were confirmed by mass spectrometry, ^1^H NMR, and ^13^C NMR, as well as by IR spectroscopy. The obtained data are consistent with those for the other derivatives [25,42].

### 3.2. Inhibitory Potency Against AChE and BuChE

All obtained compounds have been assessed as the AChE and BuChE inhibitors. Their potency was determined using the modified Ellman’s method and expressed as the half of maximal inhibitory concentration, IC_50_ [34]. Neostigmine and donepezil were applied as the reference agents. The results are collected in Table 1.

The results show that the potency of compounds is varied and some of them are characterized by good to moderate inhibitory activities. The IC_50_ values toward AChE and BuChE ranged from 76 to 98 nM or from 0.117 to 18.711 µM, respectively, for the most active derivatives. All considered compounds showed greater activity toward AChE than BuChE. Some of them were highly selective for AChE with respect to BuChE (compounds **4** and **6**), whereas compound **5** was a strong inhibitor of both cholineserases (IC_50_ = 86 nM and 117 nM). Differences in the inhibitory potency toward these enzymes result from some structural differences between the hydrophobic gorges of the active center of both enzymes [43]. 

The studied phenol–benzimidazole conjugates with the additional substituent including the modified amine group on the phenolic moiety showed a lower activity compared with the considered polyphenols and the highest activity was found for 5-nitroderivative [6,7]. This suggests that the introduction of the second hydroxyl group into the phenyl ring enhances an inhibitory potency of the compound against AChE.

### 3.3. Kinetic Studies

**Figure 2:**
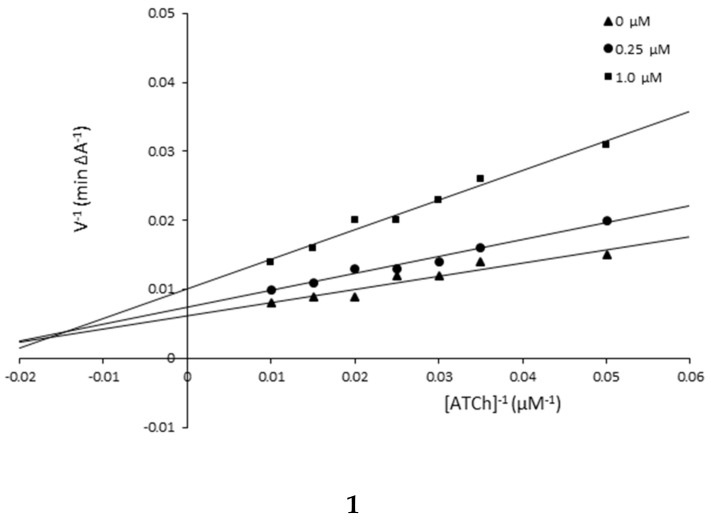

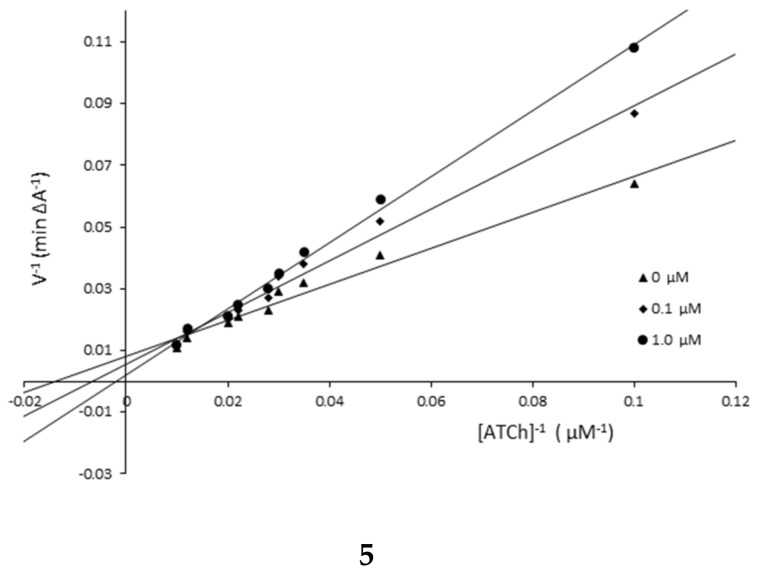
Lineweaver–Burk reciprocal plots for AChE inhibition by compounds **1** and **5**.

**Figure 3:**
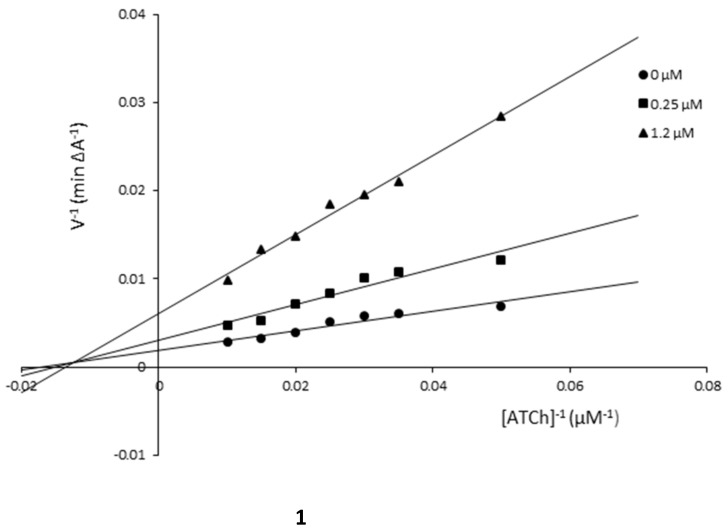

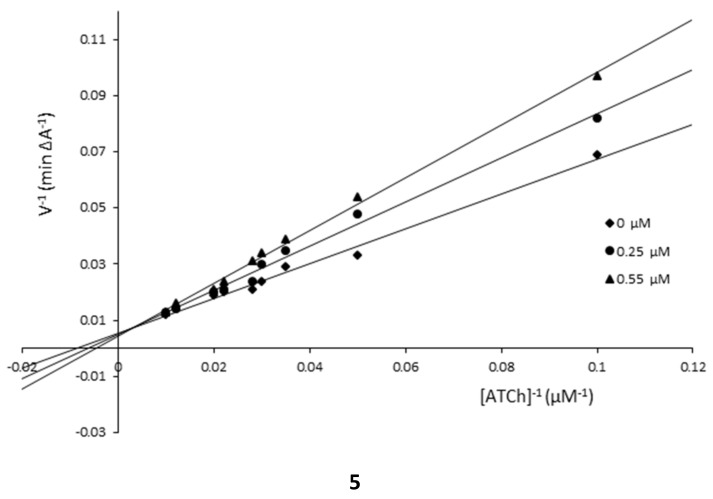
Lineweaver–Burk reciprocal plots for BuChE inhibition by compounds **1** and **5**.

The mechanism of AChE inhibition was investigated for inhibitors **1** and **5** as the most valuable compounds. The Lineweaver–Burk plots present 1/velocity versus 1/substrate concentrations in the range of 100–10 µM for compounds **5** (0.1, 1.0 µM) and **1** (0.25, 1.0 µM) and without inhibitors (0 µM) (Figure 2). Based on the Lineweaver–Burk plots analysis, the mixed type of inhibition was proven because the lines cross the same point of the coordinate system. As follows from the studies, **1** and **5** showed a different relationship: increased K_M_ and decreased υ_max_ values with higher inhibitor concentrations (Figure 2, Table 2). The mechanism of BuChE inhibition was also investigated for compounds **1** and **5** and the tests showed the same relationships as in the case of AChE (Figure 3, Table 3).

### 3.4. Inhibition of Self-induced Aβ Aggregation

**Figure 4:**
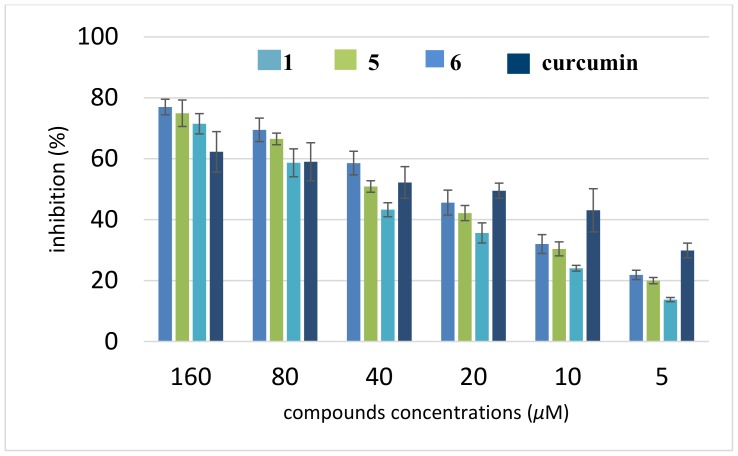
Inhibition of self-aggregation of Aβ(1–42) by compounds **1**, **5**, and **6**. The thioflavin-T fluorescence method was used and the measurements were carried out in the presence of test compounds. The mean ± SD values from three independent experiments are shown.

Nowadays, the amyloid hypothesis has become the dominating paradigm for understanding AD [44]. Aβ(1–42) is the most amyloidogenic Aβ fragment found in the AD plaques. To determine the amyloid Aβ(1–42) aggregation inhibition of the strongest AChE inhibitors (**1**, **5**, **6**), thioflavin-T (ThT) assay was performed with curcumin as the reference compound. Interestingly, all the synthesized compounds presented moderate to good potencies on the self-induced Aβ(1–42) aggregation. The data in Figure 4 show that the compounds at 5 µM inhibited Aβ(1–42) self-aggregation in the range from 14% to 22% and at 160 µM in the range from 71% to 77%. They indicate that new compounds are found to be similar or more potent than curcumin in inhibiting the self-induced Aβ42 aggregation at higher concentrations. Surprisingly, at lower concentrations, the tested compounds are less potent than the reference compound. The most effective compound is **6**, followed by **5** and **1**; their IC_50_ values were 27.04 ± 2.29, 33.07 ± 2.07, and 48.00 ± 3.43 µM, respectively (Table 4).

### 3.5. Antioxidant Potential

Reactive oxygen species are a causal factor in the process of aging and the cumulative damage has been associated with AD [45]. In this study, the DPPH radical scavenging activity of the newly synthesized compounds was estimated using the Brand-Williams method. The data showed that all tested compounds demonstrated the dose-dependent DPPH inhibition activity (Table 4). Compound **1** exhibited the lowest DPPH radical scavenging activity (0.641 ± 0.03 µM). On the contrary, in compound **8** (IC_50(Aβ(1–42))_ = 0.125 ± 0.06 µM), the radical scavenging properties were at a level similar to the used standard.

### 3.6. Molecular Modeling Studies

Three benzimidazole derivatives were taken for the molecular docking studies to present their binding mode within the enzyme and to explain the differences in the activity. Two of them were active (**1**, **5**) while one was inactive (**7**). The crystal structure of AChE from the electric ray in a complex with the reference inhibitor, tacrine, was used for docking (PDB code: 1ACJ). The docking procedure was fully validated and described previously [46].

**Figure 5:**
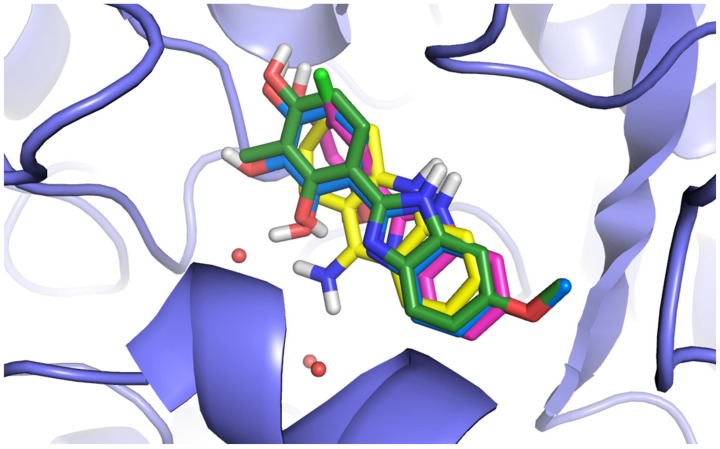
Comparison of the binding modes of selected derivatives (**1**, **5**, and **7**) and tacrine. The compounds are in the colors: **1**—pink, **5**—green, **7**—blue, and tacrine—yellow. The red spheres represent the water molecules.

All compounds presented a similar arrangement within the active site of AChE, interacting mainly with the catalytic site (Figure 5). They mimicked the binding mode of tacrine that is a mixed-type, reversible inhibitor of cholinesterase that binds close to the catalytically active serine in the active site of the enzyme [47]. The benzimidazole ring interacted by π–π stacking with the side chains of Phe330 and Trp84. The -NH fragment of this heterocyclic system created a hydrogen bond with the carbonyl group of His440. The methoxy group (compound **5**) was not engaged in any specific interactions and it seems that it was not necessary for the enzyme inhibition. The substituted phenyl moiety created π–π interactions with Trp84.

**Figure 6:**
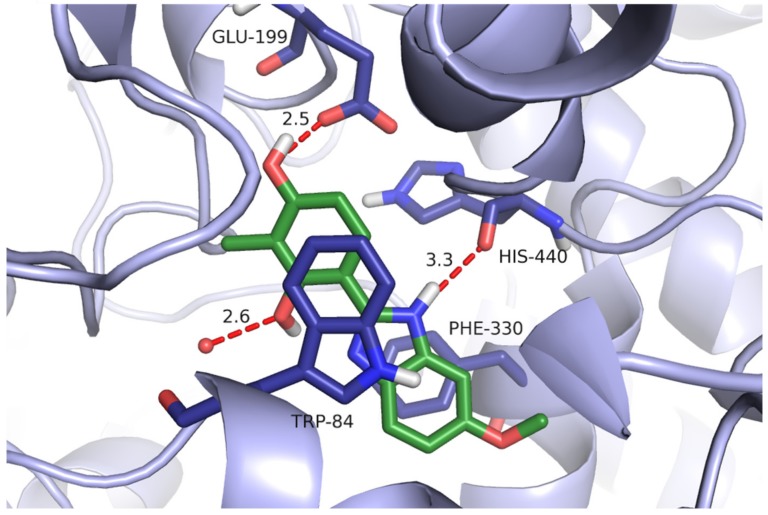
The binding mode of compound **5** within the active site of AChE. Hydrogen bonds are shown as red dashes, the distance between the donor and the acceptor for each hydrogen bond is given in Å. The red spheres represent water molecules.

The hydroxyl group in position 4 was always bound to the carboxyl group of Glu199 by the hydrogen bond, whereas the hydroxyl group in position 2 usually created the H bond with the water molecule. Comparing two active compounds with an inactive one, it could be noticed that they differed significantly from one another as far as lipophilicity is concerned. Introduction of the third hydroxyl group in position 3 (compound **7**, log P = 1.57) led to a decrease of lipophilic properties in comparison with the compounds containing an extra methyl group (chlorine atom (compound **1**, log P = 2.64 or compound **5**, log P = 2.45)). The detailed binding mode of compound **5** is presented in Figure 6.

The lower log P value (lower lipophilicity) resulted in reduction of hydrophobic and aromatic interactions, especially those of the phenyl moiety, and as a consequence, a lack of the activity. The other compound **10** with the third -OH group on the phenyl ring is also inactive. This observation is consistent with the results of our previous studies which confirmed an important role of lipophilic properties in binding to AChE [48]. Structure–activity relationship (SAR) analysis shows also an important role of lipophilicity and the polar surface area on inhibitory potency against cholinesterases. The docking simulation of benzimidazole–phenol conjugates with the additional tertiary amine group and resorcinol-1,3,4-thiadiazole hybrids proved that the compounds bound mainly with the catalytic site and confirmed an important role of hydroxyl groups in the interactions with an enzyme [7,22].

### 3.7. SAR Studies

To find the structure–activity relationships, some quantities of the compounds were estimated (Table 5). They included the lipophilicity parameter log k_w_ obtained by RP-HPLC and calculated in silico log P, the topological polar surface area (tPSA), and the molar refractivity (MR) (Table 5). The log k_w_ and S parameters obtained by the extrapolation technique are commonly used as lipophilicity descriptors [32,49,50].

To obtain better results, the previously described compound 4-(1*H*-benzimidazol-2-yl)-benzene -1,3-diol (bib-1,3-diol) as a parent compound was included in the biological screening (Table 1 and Table 5) [26]. The structure–activity elucidation shows that the presence of the third -OH group in the benzenediol moiety (compounds **7** and **10**) or -NO_2_ (**13**, **14**), particularly -CN (**11**, **12**) on the benzimidazole ring decreases significantly the inhibitory potency of the compounds against both enzymes. Introduction of the hydrophobic substituent (-Cl, -Me, -CF_3_) as well as -OMe on the heterocyclic ring or -Cl in the aryl moiety enhances biological activity of analogs in comparison to the unsubstituted bib-1,3-diol. The activity of compounds with the ethyl ester group is moderate (**8**–**10**) and it deteriorates with the addition of the hydroxyl substituent.

On the other hand, taking into account the values of the calculated descriptors (Table 5), the low tPSA as well as MR favor the activity. Small particles with a low polar surface area are preferred. However, the most active compounds are characterized by a diverse lipophilicity expressed by both log P and log k_w_. It seems that not the log P value of compounds but the location of the polar region in the molecule is important. That confirms a lack of activity for the compounds with the third polar substituent in the aryl ring (IC_50_ > 500 µM, compounds **7** and **10**).

## 4. Conclusions

In summary, a series of new benzimidazoles functionalized by 2,4-dihydroxyphenyl moiety has been obtained and evaluated as AChE and BuChE inhibitors. All compounds were more active against AChE than BuChE. Analogs **1** and **5** are the strongest inhibitors in the series (IC_50_ = 76 and 86 nM, respectively). At the same time, compound **5** is highly active against BuChE (IC_50_ = 117 nM). The kinetic studies suggest the mixed type of inhibition for both enzymes. Besides the ability to inhibit AChE and BuChE, the most active derivative also inhibited in vitro self-induced Aβ(1–42) aggregation and exhibited antioxidant properties. The compounds under consideration have the properties expected for potential drugs used in AD therapy.

The docking simulation exhibited that modeled compounds **1** and **5** create a lot of interactions with the catalytic site of AChE and they mimic the binding mode of tacrine, which confirms their high inhibitory potency. The force of binding with the enzyme and the activity depend on the lipophilicity of the compounds, molar refractivity, and topological polar surface area. Introduction of the third hydroxyl group into the phenyl ring leads to reduction of lipophilic properties (increase of tPSA) as well as of hydrophobic and aromatic interactions of enzymes, particularly those of the phenyl moiety, and as a consequence, a lack of activity is observed. The presence of the additional lipophilic substituent of both rings enhances the anticholinesterase potency of analogs compared with the parent compound.

## Figures and Tables

**Table 1:** In vitro inhibitory concentration of the compounds against AChE and BuChE (IC_50_, μM) and selectivity of the studied compounds.

Compound	AChE ^1^ IC_50_ [μM]	BuChE ^2^ IC_50_ [μM]	Selectivity for AChE ^3^
**1**	0.076 ± 0.002	4.24 ± 0.205	55.8
**2**	0.541 ± 0.036	22.69 ± 0.078	41.9
**3**	0.098 ± 0.005	14.022 ± 0.111	143.1
**4**	0.118 ± 0.009	46.025 ± 0.104	390.0
**5**	0.086 ± 0.021	0.117 ± 0.041	1.4
**6**	0.092 ± 0.002	18.711 ± 0.173	203.4
**7**	>500	>500	-
**8**	0.184 ± 0.022	25.352 ± 0.232	137.8
**9**	0.962 ± 0.023	43.855 ± 0.381	45.6
**10**	>500	>500	-
**11**	>500	>500	-
**12**	>500	>500	-
**13**	1.487 ± 0.118	106.557 ± 0.201	71.7
**14**	1.003 ± 0.196	88.363 ± 0.155	88.1
**bib-1,3-diol ^4^**	0.284 ± 0.014	31.024 ± 0.133	109.2
**neostigmine**	0.052 ± 0.003	0.068 ± 0.005	1.3
**donepezil**	0.020 ± 0.008	7.520 ± 0.200	376

^1^ IC_50_—50% inhibitory concentration (means ± SD of three independent experiments) of AChE. ^2^ IC_50_—50% inhibitory concentration (means ± SD of three experiments) of BuChE. ^3^ Selectivity for AChE = IC_50_(BuChE)/IC_50_(AChE). ^4^ 4-(1*H*-benzimidazol-2-yl)benzene-1,3-diol was previously described [27].

**Table 2:** The K_M_ and υ_max_ values at different concentrations of the inhibitor toward AChE.

Compound/Concentration [μM]	K_M_ [μM]	υ_max_ [A min^−1^]
Compound **1**		
1.0	46.41	105.26
0.25	33.16	135.14
0	28.45	153.85
Compound **5**		
1.0	487.09	120.48
0.15	147.05	175.44
0	70.33	454.55

**Table 3:** The K_M_ and **υ_max_** values at different concentrations of the inhibitor toward BuChE.

Compound/Concentration [µM]	K_M_ [μM]	υ_max_ [A min^−1^]
Compound **1**		
1.2	73.53	163.93
0.25	64.92	322.58
0	58.14	526.32
Compound **5**		
0.55	209.21	178.57
0.25	154.08	196.08
0	110.74	222.22

**Table 4:** In vitro inhibition of the self-induced aggregation IC_50(Aβ(1–42))_ and the radical scavenging effect (DPPH free radical) IC_50(DPPH)_ by the synthesized compounds.

Compound	IC_50(Aβ(1–42))_ [μM] ^1^	IC_50(DPPH)_ [μM] ^1^
**1**	48.00 ± 3.43	0.641 ± 0.03
**2**	- ^2^	0.213 ± 0.08
**5**	33.07 ± 2.07	0.627 ± 0.11
**6**	27.04 ± 2.29	0.362 ± 0.01
**8**	- ^2^	0.125 ± 0.06
**12**	- ^2^	0.209 ± 0.02
rutin	- ^2^	0.087 ± 0.04

^1^ IC_50_: 50% inhibitory concentration (means ± SD of three experiments). ^2^ Experiment was not conducted.

**Table 5:** Chromatographic parameters: log *k*_w_ (intercept) and −*S* (slope) of the linear dependences obtained by RP 18 HPLC, log *P* values, molar refractivity (MR), topological polar surface area (tPSA).

No.	log *k*_w_	−*S*	*n*	*r* ^2^	log P	tPSA [Å^2^]	MR [cm^3^·mol^−1^]
**1**	3.602	3.896	6	0.992	2.64	64.85	67.86
**2**	5.460	5.770	5	0.985	3.76	64.85	77.07
**3**	5.720	6.299	6	0.992	3.49	64.85	75.66
**4**	4.237	4.635	6	0.992	4.47	64.85	84.87
**5**	4.591	5.179	6	0.994	2.45	74.08	76.40
**6**	4.808	5.390	6	0.993	2.54	74.08	75.11
**7**	3.589	3.870	6	0.996	1.57	94,31	72.32
**8**	2.566	4.097	5	0.875	2.73	91.15	86.20
**9**	3.067	4.160	6	0.999	3.15	91.15	90.80
**10**	2.088	3.237	6	0.979	1.85	111.38	82.12
**11**	3.590	3.867	6	0.996	2.61	88.64	75.25
**12**	3.360	4.854	5	0.975	3.02	88.64	79.85
**13**	2.475	4.655	4	0.999	2.52	116.66	- ^1^
**14**	2.674	4.596	5	0.981	2.94	116.66	- ^1^
**bib-1,3-diol**	1.732	2.979	5	0.968	2.09	64.85	63.26

^1^ Parameter was not calculated.

## References

[1] Brookmeyer R., Johnson E., Ziegler-Graham K., Arrighi H.M. (2007). Forecasting the global burden of Alzheimer’s Disease. Alzheimers. Dement..

[2] Jasiecki J., Wasag B. (2019). Butyrylcholinesterase protein ends in the pathogenesis of Alzheimer’s Disease - could BCHE genotyping be helpful in Alzheimer’s therapy?. Biomolecules.

[3] Acar Cevik U., Saglik B.N., Levent S., Osmaniye D., Kaya Cavusoglu B., Ozkay Y., Kaplancikli Z.A. (2019). Synthesis and AChE-Inhibitory activity of new benzimidazole derivatives. Molecules.

[4] Refolo L.M., Fillit H.M. (2004). Drug discovery for Alzheimer’s Disease: The end of the beginning. J. Mol. Neurosci..

[5] Zhu J., Wu C.F., Li X., Wu G.S., Xie S., Hu Q.N., Deng Z., Zhu M.X., Luo H.R., Hong X. (2013). Synthesis, biological evaluation and molecular modeling of substituted 2-aminobenzimidazoles as novel inhibitors of acetylcholinesterase and butyrylcholinesterase. Bioorg. Med. Chem..

[6] Alpan A.S., Parlar S., Carlino L., Tarikogullari A.H., Alptuzun V., Gunes H.S. (2013). Synthesis, biological activity and molecular modeling studies on 1*H*-benzimidazole derivatives as acetylcholinesterase inhibitors. Bioorg. Med. Chem..

[7] Alpan A.S., Sarikaya G., Coban G., Parlar S., Armagan G., Alptuzun V. (2017). Mannich-benzimidazole derivatives as antioxidant and anticholinesterase inhibitors: Synthesis, biological evaluations, and molecular docking study. Arch. Pharm..

[8] Ozadali-Sari K., Tuylu Kucukkilinc T., Ayazgok B., Balkan A., Unsal-Tan O. (2017). Novel multi-targeted agents for Alzheimer’s disease: Synthesis, biological evaluation, and molecular modeling of novel 2-[4-(4-substitutedpiperazin-1-yl)phenyl]benzimidazoles. Bioorg. Chem..

[9] Blaszczak-Swiatkiewicz K., Sikora J., Szymanski J., Danilewicz M., Mikiciuk-Olasik E. (2016). Biological evaluation of the toxicity and the cell cycle interruption by some benzimidazole derivatives. Tumour. Biol..

[10] Yoon Y.K., Ali M.A., Wei A.C., Choon T.S., Khaw K.Y., Murugaiyah V., Osman H., Masand V.H. (2013). Synthesis, characterization, and molecular docking analysis of novel benzimidazole derivatives as cholinesterase inhibitors. Bioorg. Chem..

[11] Sari Y., Aktas A., Taslimi P., Gok Y., Gulcin I. (2018). Novel N-propylphthalimide- and 4-vinylbenzyl-substituted benzimidazole salts: Synthesis, characterization, and determination of their metal chelating effects and inhibition profiles against acetylcholinesterase and carbonic anhydrase enzymes. J. Biochem. Mol. Toxicol..

[12] Katalinic M., Macek Hrvat N., Baumann K., Morasi Pipercic S., Makaric S., Tomic S., Jovic O., Hrenar T., Milicevic A., Jelic D. (2016). A comprehensive evaluation of novel oximes in creation of butyrylcholinesterase-based nerve agent bioscavengers. Toxicol. Appl. Pharm..

[13] Aslam S., Zaib S., Ahmad M., Gardiner J.M., Ahmad A., Hameed A., Furtmann N., Gutschow M., Bajorath J., Iqbal J. (2014). Novel structural hybrids of pyrazolobenzothiazines with benzimidazoles as cholinesterase inhibitors. Eur. J. Med. Chem..

[14] Gungordu A., Sireci N., Kucukbay H., Birhanli A., Ozmen M. (2013). Evaluation of in vitro and in vivo toxic effects of newly synthesized benzimidazole-based organophosphorus compounds. Ecotoxicol. Environ. Saf..

[15] Xiang P., Zhou T., Wang L., Sun C.Y., Hu J., Zhao Y.L., Yang L. (2012). Novel benzothiazole, benzimidazole and benzoxazole derivatives as potential antitumor agents: Synthesis and preliminary in vitro biological evaluation. Molecules.

[16] Zawawi N.K., Taha M., Ahmat N., Wadood A., Ismail N.H., Rahim F., Ali M., Abdullah N., Khan K.M. (2015). Novel 2,5-disubtituted-1,3,4-oxadiazoles with benzimidazole backbone: A new class of beta-glucuronidase inhibitors and in silico studies. Bioorg. Med. Chem..

[17] Kamil A., Akhtar S., Noureen S., Saify Z.S., Jahan S., Khan K.M., Rahim F., Mushtaq N., Arif M., Perveen S. (2015). 2-(2-Pyridyl)benzimidazole analogs and their beta-glucuronidase inhibitory activity. J. Chem. Soc. Pak..

[18] Zawawi N.K., Taha M., Ahmat N., Wadood A., Ismail N.H., Rahim F., Azam S.S., Abdullah N. (2016). Benzimidazole derivatives as new alpha-glucosidase inhibitors and in silico studies. Bioorg. Chem..

[19] Arshad T., Khan K.M., Rasool N., Salar U., Hussain S., Tahir T., Ashraf M., Wadood A., Riaz M., Perveen S. (2016). Syntheses, in vitro evaluation and molecular docking studies of 5-bromo-2-aryl benzimidazoles as α-glucosidase inhibitors. Med. Chem. Res..

[20] Arshad T., Khan K.M., Rasool N., Salar U., Hussain S., Asghar H., Ashraf M., Wadood A., Riaz M., Perveen S. (2017). 5-Bromo-2-aryl benzimidazole derivatives as non-cytotoxic potential dual inhibitors of alpha-glucosidase and urease enzymes. Bioorg. Chem..

[21] Adegboye A.A., Khan K.M., Salar U., Aboaba S.A., Chigurupati S., Fatima I., Taha M., Wadood A., Mohammad J.I., Khan H. (2018). 2-Aryl benzimidazoles: Synthesis, in vitro alpha-amylase inhibitory activity, and molecular docking study. Eur. J. Med. Chem..

[22] Skrzypek A., Matysiak J., Niewiadomy A., Bajda M., Szymanski P. (2013). Synthesis and biological evaluation of 1,3,4-thiadiazole analogues as novel AChE and BuChE inhibitors. Eur. J. Med. Chem..

[23] Skrzypek A., Matysiak J., Karpinska M.M., Niewiadomy A. (2013). Synthesis and anticholinesterase activities of novel 1,3,4-thiadiazole based compounds. J. Enzym. Inhib. Med. Chem..

[24] Rzeski W., Matysiak J., Kandefer-Szerszen M. (2007). Anticancer, neuroprotective activities and computational studies of 2-amino-1,3,4-thiadiazole based compound. Bioorg. Med. Chem..

[25] Karpinska M.M., Matysiak J., Niewiadomy A., Wietrzyk J., Klopotowska D. (2012). Synthesis and biological activity of novel 4-and 6-(1-alkyl/aryl-1H-benzimidazol-2-yl)benzene-1,3-diols. Mon. Fur. Chem..

[26] Karpinka M.M., Matysiak J., Niewiadomy A. (2011). Synthesis of novel 4-(1*H*-benzimidazol-2-yl)benzene-1,3-diols and their cytotoxic activity against human cancer cell lines. Arch. Pharm. Res..

[27] Tavman A., Birteksoz A.S. (2009). Spectral characterization and antimicrobial activity of 4-(5-H/Me/Cl/NO_2_-1*H*-benzimidazol-2-yl)-benzene-1,3-diols and some metal complexes. Revi. Inorg. Chem..

[28] Tavman A., Cinarli A., Gurbuz D., Birteksoz A.S. (2012). Synthesis, characterization and antimicrobial activity of 2-(5-H/Me/F/Cl/NO_2_-1*H*-benzimidazol-2-yl)-benzene-1,4-diols and some transition metal complexes. J. Iran. Chem. Soc..

[29] Perry E.K., Tomlinson B.E., Blessed G., Bergmann K., Gibson P.H., Perry R.H. (1978). Correlation of cholinergic abnormalities with senile plaques and mental test scores in senile dementia. Br. Med. J..

[30] Wang X., Wang W., Li L., Perry G., Lee H.G., Zhu X. (2014). Oxidative stress and mitochondrial dysfunction in Alzheimer’s disease. Biochim. Biophys. Acta..

[31] https://scifinder.cas.org.

[32] Soczewinski E., Axelwach C. (1962). Relation between composition of certain ternary 2-phase solvent systems and Rm values. J. Chromatogr..

[33] Rozylo J.K., Zabinska A., Matysiak J., Niewiadomy A. (1999). Reversed-phase thin-layer chromatography with different stationary phases in studies of quantitative structure-biological activity relationship of new antimycotic compounds. J. Aoac. Int..

[34] Ellman G.L., Courtney K.D., Andres V., Feather-Stone R.M. (1961). A new and rapid colorimetric determination of acetylcholinesterase activity. Biochem. Pharm..

[35] Wang Z., Wang Y., Wang B., Li W., Huang L., Li X. (2015). Design, Synthesis, and evaluation of orally available Clioquinol-Moracin M hybrids as multitarget-directed ligands for cognitive improvement in a rat model of neurodegeneration in Alzheimer’s Disease. J. Med. Chem..

[36] Czarnecka K., Girek M., Krecisz P., Skibinski R., Latka K., Jonczyk J., Bajda M., Kabzinski J., Majsterek I., Szymczyk P. (2019). Discovery of new cyclopentaquinoline analogues as multifunctional agents for the treatment of Alzheimer’s Disease. Int. J. Mol. Sci..

[37] Hamdy N.A., Anwar M.M., Abu-Zied K.M., Awad H.M. (2013). Synthesis, tumor inhibitory and antioxidant activity of new polyfunctionally 2-substituted 5,6,7,8-tetrahydronaphthalene derivatives containing pyridine, thioxopyridine and pyrazolopyridine moieties. Acta. Pol. Pharm..

[38] Michael H.N., Awad H.M., El-Sayed N.H., Pare P.W. (2010). Chemical and antioxidant investigations: Norfolk pine needles (Araucaria excelsa). Pharm. Biol..

[39] Ghose A.K., Crippen G.M. (1987). Atomic physicochemical parameters for three-dimensional-structure-directed quantitative structure-activity relationships. 2. Modeling dispersive and hydrophobic interactions. J. Chem. Inf. Comput. Sci..

[40] Ertl P., Rohde B., Selzer P. (2000). Fast calculation of molecular polar surface area as a sum of fragment-based contributions and its application to the prediction of drug transport properties. J. Med. Chem..

[41] Matysiak J., Niewiadomy A. (2006). Application of sulfinyl bis(2,4-dihydroxythiobenzoyl) in the synthesis of *N*-substituted 2-amino-5-(2,4-dihydroxyphenyl)-1,3,4-thiadiazoles. Synth. Commun..

[42] Matysiak J., Niewiadomy A. (2003). Synthesis and antimycotic activity of *N*-azolyl-2,4-dihydroxythiobenzamides. Bioorg. Med. Chem..

[43] Cheung J., Rudolph M.J., Burshteyn F., Cassidy M.S., Gary E.N., Love J., Franklin M.C., Height J.J. (2012). Structures of human acetylcholinesterase in complex with pharmacologically important ligands. J. Med. Chem..

[44] Kepp K.P. (2017). Ten challenges of the amyloid hypothesis of Alzheimer’s disease. J. Alzheimers. Dis..

[45] Drummond N.J., Davies N.O., Lovett J.E., Miller M.R., Cook G., Becker T., Becker C.G., McPhail D.B., Kunath T. (2017). A synthetic cell permeable antioxidant protects neurons against acute oxidative stress. Sci. Rep..

[46] Bajda M., Wieckowska A., Hebda M., Guzior N., Sotriffer C.A., Malawska B. (2013). Structure-based search for new inhibitors of cholinesterases. Int. J. Mol. Sci..

[47] Catalan R.E., Martinez A.M., Aragones M.D., Miguel B.G., Hernandez F., Cruz E. (1993). Tetrahydroaminoacridine affects the cholinergic function of blood-brain barrier. Life. Sci..

[48] Godyn J., Hebda M., Wieckowska A., Wieckowski K., Malawska B., Bajda M. (2017). Lipophilic properties of anti-Alzheimer’s agents determined by micellar electrokinetic chromatography and reversed-phase thin-layer chromatography. Electrophoresis.

[49] Janicka M., Kwietniewski L., Matysiak J. (2000). A new method for estimating log k(w) values and solute biological activity. Jpc-J. Planar. Chromat..

[50] Niewiadomy A., Zabinska A., Matysiak J., Rozylo J.K. (1997). Influence of modifier and molecular structure of some dihydroxythiobenzanilides on retention in reversed-phase high-performance thin-layer chromatography. J. Chromatogr. A.

